# L-proline/cholesterol and diosgenin based thiourea cooperative system for the direct asymmetric aldol reaction in the presence of water

**DOI:** 10.3906/kim-2003-36

**Published:** 2020-10-26

**Authors:** Serkan EYMUR, Enis TAŞCI, Arzu UYANIK, Mustafa YILMAZ

**Affiliations:** 1 Department of Energy Systems Engineering, Faculty of Engineering, Giresun University, Giresun Turkey; 2 Vocational School of Health Services, Giresun University, Giresun Turkey; 3 Department of Chemistry, Faculty of Science, Selçuk University, Konya Turkey

**Keywords:** Aldol, organocatalyst, proline, asymmetric synthesis

## Abstract

A series of cholesterol and based hydrophobic urea and thiourea compounds were synthesized and successfully used as a cocatalyst for ﻿L-proline catalyzed aldol reactions in the presence of water. The anticonfigured products were obtained with good yields (up to 94%), high diastereoselectivities (up to 95:5), and high enantiomeric excesses (up to 93% *ee* ). ﻿The successful results for catalytic efficiency of L-proline in the presence of water reveal the importance of the hydrophobic nature of cholesterol and diosgenin parts of thiourea on the reactivity and selectivity in the presence of water.

## 1. Introduction

Direct asymmetric aldol reaction is one of the most effective strategies inspired by nature for stereoselective carbon-carbon bond-forming reactions in synthetic organic chemistry [1–3]. Following the pioneering studies on L-proline catalyzed direct asymmetric aldol reactions by List et al. in the early 2000s [4], much effort has been made to develop the effective metal-free small organic molecules as organocatalytic systems for direct aldol reactions, which generally involves structural modifications of catalysts and optimization of the reaction conditions. However, classical organocatalyst synthesis requires a painstaking strategy and can involve challenging synthesis steps. Since only one catalyst is used in these organocatalyst samples, reactivities and selectivity are also expected to be limited [5,6]. Most recently, there has been considerable interest in employing self-assembled organocatalysts in catalytic asymmetric reactions [5,6]. The use of such self-assembled organocatalytic systems has advantages over the conventional organocatalysts; such as (i) the structure of self-assembled organocatalysts is easy for modification and optimization, (ii) it is very easy to create a large catalyst library by changing selected suitable additives. We have successfully investigated and determined that the self-assembled proline-thioureasupramolecular complex was an efficient organocatalyst for a direct enantioselective aldol reaction in nonpolar solvents such as hexane [7]. Also, researchers have shown that the use of suitable additives, such as water [8–10], chiral alcohols [11,12], thioureas [13–17], thiouronium salts [18], imidazolium salts [19], and guanidinium salts [20] has been documented as a powerful method to accelerate the rate of reaction and improve the stereoselectivity of aldol reactions. 

Due to environmental concerns, reactions using water as a solvent have recently received significant attention from a wide range of synthetic chemists [21,22]. L-proline is known as an efficient catalyst for direct aldol reactions in generally polar organic solvents [23,24]. Nevertheless, the high polarity of these organic solvents has continued to be a major problem from a viewpoint of green chemistry. While L-proline was known to be an inefficient catalyst in water, Barbas and Hayashi’s groups revealed that hydrophobic proline derivatives could effectively catalyze asymmetric aldol reactions in the presence of water [25–28]. This concept opens a new avenue for the development of new hydrophobic water-compatible organocatalysts [29–31]. Inspired by the introduction of a suitable hydrophobic moiety into organocatalytic systems, we recently showed that the use of calixarene-linked thiourea as a hydrophobic cocatalyst that has good H-bonding ability in supramolecular interactions increased the yield and selectivity of the catalytic asymmetric aldol reactions in the presence of water [15]. 

Cholesterol and diosgenin are known to be essential components of mammalian cellular membranes; they provide the membranes with improved lipophilic characteristics over linear alkyl chains [32]. When one considers the natural amphiphilic structures of cholesterol and diosgenin molecules, it is surprising that their use as a hydrophobic part of water-compatible organocatalysts has only received limited attention [33]. 

We are still keenly interested in improving the efficiency of L-proline catalyzed aldol reactions in water. Previously, we have successfully established that calixarene-linked thiourea was an effective cocatalyst for the highly stereoselective intermolecular aldol reaction in the presence of water [15]. The results clearly confirmed that the hydrophobic calix[4]arene part of thiourea has a positive effect on both reactivity and stereoselectivity. Since the development of suitable cocatalysts that form assemblies with proline, direct aldol reactions in the presence of water are still desirable; herein we turned our attention to the synthesis of new thiourea and urea derivatives bearing cholesterol and diosgenin moieties as hydrophobic motifs. The aim of this study was to develop a small cocatalyst library of cholesterol and diosgenin based (thio)ureas which can self-assemble with L-proline to catalyze the asymmetric aldol reaction of cyclohexanone and aromatic aldehydes in the presence of water.

## 2. Results and discussion

**Scheme 1:**
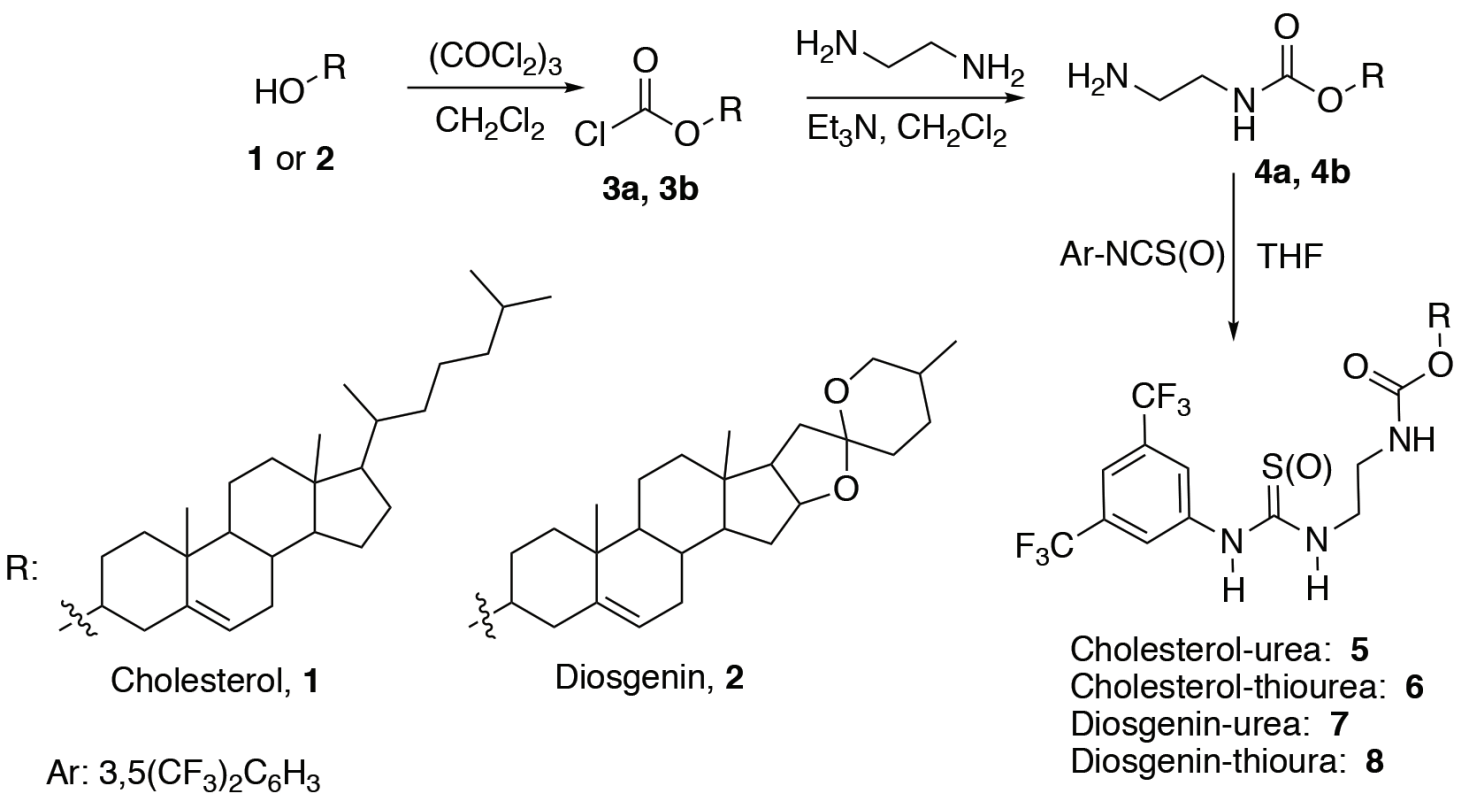
Synthesis of urea derivatives 5–8.

The synthetic route to a series of thiourea catalysts is illustrated in Scheme 1. Carbamate derivatives 4a and 4b were synthesized according to a published procedure [33,34]. First, the reaction of cholesterol (1) and diosgenin (2) with triphosgene gave chloroformate derivatives 3a and 3b, respectively. Then, the compounds 3a and 3b were reacted with ﻿ethylenediamine, and the corresponding carbamate derivatives 4a and 4b were obtained. Finally, these carbamate derivatives were converted into their thiourea derivatives 6 and 8 by treatment with phenyl isothiocyanate. A similar synthetic route was used to prepare the urea derivatives 5 and 7. The structures of compounds 3–8 were fully identified by using ^1^H and ^13^C NMR and mass spectroscopy.

With the desired cholesterol and diosgenin (thio)urea derivatives in hand, we next studied the possibility of using these thiourea derivatives 5–8 as new cocatalysts in the L-proline catalyzed aldol reaction in the presence of water. As a test reaction, the aldol reaction of cyclohexanone and p-nitrobenzaldehyde was conducted in the presence of water. As shown in Table 1, all the examined cocatalysts 5–8 showed similar levels of catalytic efficiencies, with high conversions, diastereo- and enantioselectivities. ﻿The best result in terms of selectivity was obtained by using L-proline (10 mol %) / cholesterol-based thiourea 6 (10 mol %), and the reaction furnished the expected product in nearly full conversion with 93% *ee* (entry 2, Table 1). Next, we screened the amount of water and found that our system showed high catalytic efficiency in 0.250 mL of water. We observed that an ﻿increase in the amount of water (0.5 mL) decreases the enantioselectivity of the aldol product (entry 5, Table 1). Reducing the amount of water to 0.125 mL also reduced enantioselectivity (entry 6, Table 1). Next, we examined the effect of additives on the enantioselectivity of the reaction. However, no enhancement in selectivity was observed with different acidic additives (entries 7–9; Table 1). ﻿L-proline was found to not help the reaction (entry 12; Table 1). It was also found that cholesterol-thiourea (6) was not effective when L-proline was not used (entry 11, Table 1).

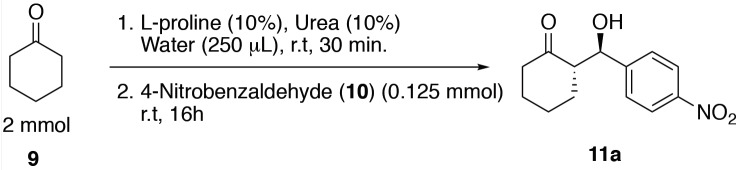



**Table 1:** Effect of cholesterol and diosgenin based urea and thiourea compounds 5-8 on proline catalyzed direct aldol reaction.

Entry	Co-catalyst	Additive	Conv. (%)a	anti:syna	eeb (%)
1	5	-	> 99	81:19	93
2	6	-	> 99	95:5	93
3	7	-	79	89:11	90
4	8	-	94	91:9	93
5c	6	-	85	90:10	89
6d	6	-	78	89:11	90
7	6	ClCH_2_COOH	91	88:12	90
8	6	CH_3_COOH	93	89:11	71
9	6	PhCOOH	94	90:10	89
10e	6	-	72	89:11	71
11f	6	-	< 5	N.D.g	N.D.
12	-	-	< 5	N.D.	N.D.

a: Determined by ^1^H NMR analysis of crude reaction mixture. b: Determined by chiral HPLC analysis. c: 0.5 mL water was used. d: 0.125 mL water was used. e: 4.0 equiv. of ketone was used. f: L-proline was not used. g: N.D. = not determined.

With these optimal reaction conditions in hand, we next studied the substrate scope of the aldol reaction of different aldehydes with cyclohexanone, and the results are presented in Table 2. The results indicated that L-proline-cholesterol based thiourea 6 host-guest complex can catalyze the aldol reaction very well in the presence of water. As seen, various substituted aromatic aldehydes with electron-withdrawing groups can be tolerated. The reaction can provide aldol products 11a to 11l in good yields with moderate to good enantioselectivity and diastereoselectivity.

Among the substituted benzaldehydes, the best enantioselectivities were obtained with the p-nitrobenzaldehyde and p-chlorobenzaldehyde, giving high enantioselectivities with 93% and 91% *ee* , respectively (entries 1 and 4, Table 2). Also, the reaction is tolerant of other p-substituted benzaldehydes, which affords the aldol product with moderate to good enantioselectivities ranging from 83% to 90%. Besides, the reaction allowed electron-withdrawing substituents at the o- and m- positions of the phenyl ring (entries 2, 3, 5, and 6, Table 2). Anisaldehyde, an electron-rich aromatic aldehyde, reacted with cyclohexanone, and the corresponding antialdol product 11k was obtained in only low yields and low enantioselectivity (36% ee) (entry 11, Table 2). We also found that cyclopentanone underwent a smooth reaction with p-nitrobenzaldehyde to give mainly the syn-product in high yield with low enantioselectivities (entry 12, Table 2).

In conclusion, we have synthesized a series of novel cholesterol-(thio)urea and diosgenin-(thio)urea conjugates as a cocatalyst that can self-assemble with L-proline to catalyze the direct aldol reactions of cyclohexanone with benzaldehyde derivatives in the presence of water. Under the optimum reaction conditions, the reaction of electron-deficient aromatic aldehydes with cyclohexanone gave anticonfigured aldol products in moderate to high *ee* values (up to 93% *ee* ) in the presence of water. The successful results for catalytic efficiency of L-proline indicate the importance of the hydrophobic nature of cholesterol and diosgenin parts of thiourea on both the reactivity and selectivity in the presence of water at room temperature.

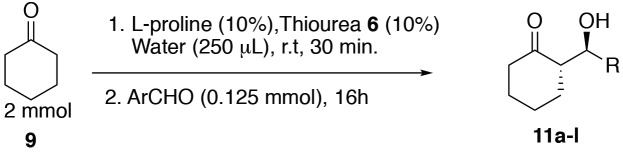



**Table 2:** Scope of aromatic aldehydes.

Entry	Aldehyde (R)	Yield (%)a	anti:syn	ee (%)
1	4-NO_2_Ph	94	95:5	93
2	3-NO_2_Ph	93	84:16	90
3	2-NO_2_Ph	65	90:10	90
4	4-ClPh	73	84:16	91
5	3-ClPh	87	90:10	88
6	2-ClPh	62	87:13	85
7	4-BrPh	75	90:10	90
8	4-FPh	90	85:15	87
9	4-CNPh	83	88:12	84
10	4-CF_3_Ph	86	85:15	83
11	4-MeOPh	44	85:15	36
12b	4-NO_2_Ph	88	42:58	68

a. Yields of isolated aldol product. b. Cyclopentanone was used.

## 3. Materials

### 3.1. General

All reagents were used as received without purification. ﻿^1^H NMR(400 MHz) and ^13^C NMR(100 MHz) spectra were taken on a Bruker Avance 400 spectrometer. TMS was used as internal standard. Precoated Merck 60 F254TLC plates were used for thin layer chromatography (TLC). ﻿Flash column chromatography was performed using silica gel (60-mesh; Merck). The ^1^H NMR, ^13^C NMR, and HRMS spectra for compounds 5–8 and HPLC chromatograms of compounds 11a–11l can be found under the ‘supplementary information’ given at the end of the article.

### 3.2. Synthesis and characterization﻿

#### 3.2.1. Synthesis of catalysts 5–8

Compound 4a (or compound 4b) (1.5 mmol) was dissolved in dry CH_2_Cl_2_(30 mL) in a round-bottom flask and cooled to ice salt temperature. Then, 3,5-bis(trifluoromethyl)phenyl isocyanate (or 3,5-bis(trifluoromethyl)phenyl isothiocyanate) (1.65 mmol) was added through a syringe and the mixture was stirred at ambient temperature for 24 h to provide a precipitate. This precipitate was washed with n-hexane and then filtered and dried in a vacuum. The crude product was crystallized with petroleum ether-methanol. 

**3.2.1.1.** Compound 5; 74% yield; white solid, mp: 213–218 °C; ^1^H NMR (CDCl3) δ: 0.62 (s, 3H), 0.75–1.60 (m, 33H), 1.70–2.00 (m, 5H), 2.10–2.30 (m, 2H), 2.98–3.35 (m, 4H), 4.30 (m, ^1^H), 5.25 (broad, ^1^H), 6.40 (broad, ^1^H), 7.02 (s, ^1^H), 7.46 (s, ^1^H), 8.07 (s, 2H), 9.39 (broad, ^1^H); ^13^C NMR (CDCl3) δ: 11.7, 18.6, 19.0, 20.9, 22.4, 22.6, 23.7, 24.2, 27.9, 28.0, 28.1, 31.8, 36.7, 35.7, 36.1, 36.4, 36.9, 38.4, 39.4, 39.7, 39.8, 40.7, 42.2, 50.0, 56.1, 56.6, 74.8, 114.8, 117.8, 121.9, 122.5, 124.7, 131.4, 131.8, 132.1, 132.4, 139.6, 141.4, 156.1, 157.6;﻿LCMS (ESI^+^): C_39_H_55_F_6_N_3_O_3_, calculated value 727.4100 ([M+H]^+^); experimental 728.4210 ([M+H]^+^). 

**3.2.1.2.** Compound 6; 70% yield, white solid, mp:68–70 °C; ^1^H NMR (CDCl3) δ: 0.69 (s, 3H), 0.80–2.10 (m, 38H), 2.20–2.40 (m, 2H) 3.15–3.55 (m, 2H), 3.60–4.00 (m, 2H), 4.20–4.60 (m, ^1^H), 5.11 (s, ^1^H), 5.25–5.40 (m, ^1^H), 7.43–8.17 (m, 5H); ^13^C NMR (CDCl3) δ 11.8, 18.7, 19.2, 21.0, 22.6, 22.8, 23.9, 24.3, 28.0, 28.2, 31.8, 35.8, 36.2, 36.5, 36.8, 38.4, 39.5, 39.7, 42.3, 49.9, 56.2, 56.6, 75.7, 118.9, 120.4, 120.5, 121.1, 121.6, 122.8, 123.8, 124.0, 124.4, 125.8, 127.1, 133.2, 133.6, 134.0, 139.3, 158.1, 181.3; ﻿LCMS (ESI^+^): C_39_H_55_F_6_N_3_O_2_S, calculated value 744.3900 ([M+H]^+^); experimental 744.4027 ([M+H]^+^). 

**3.2.1.3.** Compound 7; 75% yield, white solid; mp: 165–169 °C; ^1^H NMR (CDCl3) δ: 0.76–0.83 (m, 6H), 0.85–2.10 (m, 30H), 2.23–2.50 (m, 2H), 3.30–3.55 (m, 4H), 4.38–4.58 (m, 2H), 5.15–5.35 (m, 2H), 5.92 (s, ^1^H), 7.54 (s, ^1^H), 7.90 (s, 2H), 8.17 (s, ^1^H); ^13^C NMR (CDCl3) δ 14.1, 14.5, 16.2, 17.1, 19.2, 19.3, 20.8, 22.6, 28.0, 28.8, 30.3, 31.3, 31.4, 31.6, 31.8, 31.9, 36.6, 36.8, 39.7, 40.2, 41.6, 49.9, 56.4, 62.1, 66.9, 75.5, 80.8, 109.4, 118.3, 118.5, 121.8, 122.5, 124.5, 132.0, 132.3, 139.4, 140.8, 155.8, 158.0; ﻿LCMS (ESI^+^): C_39_H_51_F_6_N_3_O_5_ calculated value 756.3733 ([M+H]^+^); experimental 756.3761 ([M+H]^+^).

**3.2.1.4.** Compound 8; 71% yield, white solid; mp: 75–78 °C; ^1^H NMR (CDCl3) δ: 0.77–0.83 (m, 6H), 0.90–2.05 (m, 29H), 2.20–2.45 (m, 2H), 3.25–3.55 (m, 4H), 3.77 (broad, ^1^H), 4.20–4.55 (m, 2H), 5.05–5.40 (m, 2H), 7.40–8.10 (m, 5H); ^13^C NMR (CDCl3) δ: 14.6, 16.4, 17.3, 19.4, 20.9, 28.1, 28.9, 30.4, 31.5, 32.0, 36.8, 36.9, 38.5, 39.8, 40.4, 41.7, 50.0, 56.5, 62.1, 67.0, 80.9, 109.2, 118.5, 119.0, 120.6, 121.1, 121.3, 121.6, 121.8, 122.6, 124.0, 124.2, 124.5, 125.9, 126.7, 133.0, 133.4, 133.7, 134.0, 134.2, 139.5, 141.0, 158.2, 181.4; LCMS (ESI^+^): C_39_H_51_F_6_N_3_O_4_S calculated value 772.3504 ([M+H]^+^); experimental 772.3377 ([M+H]^+^). 

#### 3.2.2. ﻿General procedure for the synthesis of aldol products (11a–11i)

A mixture of L-proline (0.025 mmol), cholesterol based thiourea 6 (0.0125 mmol), cyclohexanone (0.75 mmol), and 0.25 mL water was stirred for 30 min at ambient temperature. Then, aldehyde (0.25 mmol) was added and the reaction mixture was left stirring until no further conversion is seen by TLC. The reaction mixture was treated with saturated aqueous NH_4_Cl solution and extracted with ethyl acetate. The organic layer was washed with brine, dried over anhydrous MgSO_4_, and concentrated. The product was purified with column chromatography over silica gel using ethyl acetate-hexane as an eluent. 

**3.2.2.1.** (S)-2-[(R)-hydroxy(p-nitrophenyl]methyl)-cyclohexanone (11a) [35,36]. Yield: 94%, anti/syn: 95:5, ee: 93%. HPLC: Chiralpak OD-H, iPrOH/hexane 20:80,0.5 mL/min, l = 254 nm, t_r_(min): 29.7 (minor), 38.6 (major). 

**3.2.2.2.** (S)-2-[(R)-hydroxy(m-nitrophenyl)methyl]-cyclohexanone(11b) [36]. Yield: 93%, anti/syn: 84:16, ee: 90%. HPLC: Chiralpak OD-H, iPrOH/hexane 20:80,0.5 mL/min,l = 254 nm, t_r_ (min): 22.7 (major), 27.6 (minor). 

**3.2.2.3.** ( *S* )-2-[( *R* )-hydroxy( *o* -nitrophenyl)methyl]-cyclohexanone (11c) [36]. Yield: 65%, anti/syn: 90:10, *ee* : 90%. HPLC: Chiralpak OD-H, *i* PrOH/hexane 20:80,0.5 mL/min, l= 254 nm, t_r_ (min): 24.9 (major), 26.8 (minor).

**3.2.2.4.** (S)-2-[(R)-hydroxy(p-chlorophenyl)methyl]-cyclohexanone (11d) [37]. Yield: 73%, anti/syn: 84:16, ee: 91%. HPLC: Chiralpak OD-H, iPrOH/hexane 5:95,1.0 mL/min, l = 220 nm, t_r_ (min): 22.0 (minor), 26.0(major). 

**3.2.2.5.** (S)-2-[(R)-hydroxy(m-chlorophenyl)methyl]-cyclohexanone (11e) [38]. Yield: 87%, anti/syn: 90:10, ee: 88%. HPLC: Chiralpak OD-H, iPrOH/hexane 4:96,1.0 mL/min, l = 220 nm, t_r_ (min): 23.7 (major), 26.4 (minor).

**3.2.2.6.** (S)-2-[(R)-hydroxy(o-chlorophenyl)methyl]-cyclohexanone (11f) [38]. Yield: 62%, anti/syn: 87:13, ee: 85%. HPLC: Chiralpak OD-H, iPrOH/hexane 5:95,1.0 mL/min, l = 220 nm, t_r_ (min): 17.5(minor), 19.9 (major).

**3.2.2.7.** (S)-2-[(R)-hydroxy(p-bromophenyl)methyl]-cyclohexanone (11g) [35,36]. Yield: 75%, anti/syn: 90:10, ee: 90%. HPLC: Chiralpak OD-H, iPrOH/hexane 10:90,0.5 mL/min, l = 220 nm, t_r_ (min): 31.4 (minor) ,36.5 (major).

**3.2.2.8.** (S)-2-[(R)-hydroxy(p-fluorophenyl)methyl]-cyclohexanone (11h) [37]. Yield: 90%, anti/syn: 85:15, ee: 87%. HPLC: Chiralpak OD-H, iPrOH/hexane 5:95,0.5 mL/min, l = 254 nm, t_r_ (min): 43.5 (minor), 49.4 (major).

**3.2.2.9.** (S)-2-[(R)-hydroxy(p-cyanophenyl)methyl]-cyclohexanone(11i) [38]. Yield: 83%, anti/syn:88:12, ee: 84%. HPLC: Chiralpak OD-H, iPrOH/hexane 5:95,1.0 mL/min, l = 220 nm, t_r_ (min): 27.8 (minor), 35.6 (major).

**3.2.2.10.** (S)-2-[(R)-hydroxy(p-trifluoromethhylphenyl)methyl]cyclohexanone (11j) [38]. Yield: 86%, anti/syn: 85:15, ee: 83%. HPLC: Chiralpak OD-H, iPrOH/hexane 5:95,1.0 mL/min, l = 254 nm, t_r_ (min): 17.2(minor), 22.3 (major).

**3.2.2.11.** (S)-2-[(R)-hydroxy(p-methoxyphenyl)methyl]-cyclohexanone (11k) [35,36]. Yield: 44%, anti/syn: 85:15, ee: 36%. HPLC: Chiralpak AD-H, iPrOH/hexane 10:90,0.5 mL/min, l = 254 nm, t_r_ (min): 27.9 (major), 39.9 (minor).

**3.2.2.12.** (R)-2-[(R)-hydroxy(p-nitrophenyl)methyl]-cyclopentanone (11l) [39]. Yield: 88%, anti/syn: 42:58, ee: 68%. HPLC: Chiralpak AD-H, iPrOH/hexane 5:95,0.5 mL/min, l = 210 nm, t_r_ (min): 106.8(minor), 113.3(major).

## References

[ref1] (2013). Modern Methods in Stereoselective Aldol Reactions. Weinheim, Germany: Wiley-VCH,.

[ref2] (2010). The direct catalytic asymmetric aldol reaction. Chemical Society Reviews.

[ref3] (2018). Catalytic enantioselective aldol reactions. Chemical Society Reviews.

[ref4] (2000). Proline-catalyzed direct asymmetric aldol reactions. Journal of the American Chemical Society.

[ref5] (2017). Asymmetric supramolecular organocatalysis: a complementary upgrade to organocatalysis. European Journal of Organic Chemistry.

[ref6] (2016). Additive effects on asymmetric catalysis. ﻿Chemical Reviews.

[ref7] (2009). Direct enantioselective aldol reactions catalyzed by a proline-thiourea host-guest complex. Chemical Communications.

[ref8] (2005). Primary amine catalyzed direct asymmetric aldol reaction assisted by water. Tetrahedron-Asymmetry.

[ref9] (2004). Proline-catalyzed ketone-aldehyde aldol reactions are accelerated by water. Synlett.

[ref10] (2006). Effect of additives on the proline-catalyzed ketone-aldehyde aldol reactions. Tetrahedron.

[ref11] (2006). Chiral diols: A new class of additives for direct aldol reaction catalyzed by L-proline. Journal of Organic Chemistry.

[ref12] (2006). - or (S)-bi-2-naphthol assisted, L-proline catalyzed direct aldol reaction. Tetrahedron-Asymmetry.

[ref13] (2009). Highly enantio- and diastereoselective organocatalytic desymmetrization of prochiral cyclohexanones by simple direct aldol reaction catalyzed by proline. Chemistry – A European Journal.

[ref14] (2010). Substrate-dependent nonlinear effects in proline–thiourea-catalyzed aldol reactions: unraveling the role of the thiourea co-catalyst. European Journal.

[ref15] (2014). Proline-calixarene thiourea host-guest complex catalyzed enantioselective aldol reactions: from nonpolar solvents to the presence of water. Tetrahedron-Asymmetry.

[ref16] (2013). Self-assembly of an organocatalyst for the enantioselective synthesis of Michael adducts and alpha-aminoxy alcohols in a nonpolar medium. Tetrahedron-Asymmetry.

[ref17] (2013). Synthesis of a bipyridine-derived achiral thiourea trifluoromethanesulfonic acid salt and its application as an additive in organocatalytic asymmetric reactions. Tetrahedron Letters.

[ref18] (2014). Direct asymmetric aldol reaction co-catalyzed by L-proline and isothiouronium salts. Tetrahedron Letters.

[ref19] (2013). Chemoenzymatic synthesis of optically active 2-(2′- or 4′-substituted-^1^H-imidazol-1-yl)cycloalkanols: chiral additives for (l)-proline. Catalysis Science & Technology.

[ref20] (2011). Direct aldol reactions catalyzed by a heterogeneous guanidinium salt/proline system under solvent-free conditions. Organic Letters.

[ref21] (2011). Catalytic asymmetric carbon-carbon bond-forming reactions in aqueous media. Tetrahedron-Asymmetry.

[ref22] (2005). Organic reactions in aqueous media with a focus on carbon−carbon bond formations:  a decade update. Chemical Reviews.

[ref23] (2008). Seebach’s oxazolidinone is a good catalyst for aldol reactions. Tetrahedron Letters.

[ref24] (2004). New mechanistic studies on the proline-catalyzed aldol reaction. In: Proceedings of the National Academy of Sciences of the United States of America.

[ref25] (2006). Combined proline–surfactant organocatalyst for the highly diastereo- and enantioselective aqueous direct cross-aldol reaction of aldehydes. Angewandte Chemie International Edition.

[ref26] (2006). Highly diastereo- and enantioselective direct aldol reactions in water. Angewandte Chemie International Edition.

[ref27] (2006). Organocatalytic direct asymmetric aldol reactions in water. Journal of the American Chemical Society.

[ref28] (2006). Organocatalytic direct michael reaction of ketones and aldehydes with β-nitrostyrene in brine. Journal of the American Chemical Society.

[ref29] (2013). Water in Organocatalytic Reactions. Comprehensive Enantioselective Organocatalysis.

[ref30] (2015). Water: the most versatile and nature’s friendly media in asymmetric organocatalyzed direct aldol reactions. Tetrahedron-Asymmetry.

[ref31] (2016). Water in asymmetric organocatalytic systems: a global perspective. Organic & Biomolecular Chemistry.

[ref32] (2019). Organocatalysis in aqueous media. Nature Reviews Chemistry.

[ref33] (2013). Water promoted enantioselective aldol reaction by proline-cholesterol and -diosgenin based amphiphilic organocatalysts. Tetrahedron.

[ref34] (2012). Perylen-3-ylmethyl: fluorescent photoremovable protecting group (FPRPG) for carboxylic acids and alcohols. Tetrahedron.

[ref35] (2008). Proline-based dipeptides with two amide units as organocatalyst for the asymmetric aldol reaction of cyclohexanone with aldehydes. Tetrahedron.

[ref36] (2008). Short α/β-peptides as catalysts for intra- and intermolecular aldol reactions. The Journal of Organic Chemistry.

[ref37] (2007). Highly enantioselective organocatalytic direct aldol reaction in an aqueous medium. Organic Letters.

[ref38] (2009). Highly diastereo- and enantioselective direct aldol reactions by 4-tert-butyldimethylsiloxy-substituted organocatalysts derived from N-prolylsulfonamides in water. Tetrahedron-Asymmetry.

[ref39] (2005). A highly efficient organocatalyst for direct aldol reactions of ketones with aldedydes. Journal of the American Chemical Society.

